# Supplementary material for: DMRforPairs: identifying Differentially Methylated Regions between unique samples using array based methylation profiles
Source: BMC Bioinformatics. 2014 May 15;15:141. doi: 10.1186/1471-2105-15-141 (PMC4046028; doi:10.1186/1471-2105-15-141)
Supplement: Additional file 2 — DMRforPairs output for the comparison of A431-MCF7 and NA17018-NA17105. Please start from the HTML files in each folder. Available via the BMC Bioinformatics website. [file 1471-2105-15-141-S2.zip › 1394847754114233_MOESM2_ESM/A431_MCF7/figures/12439.pdf]

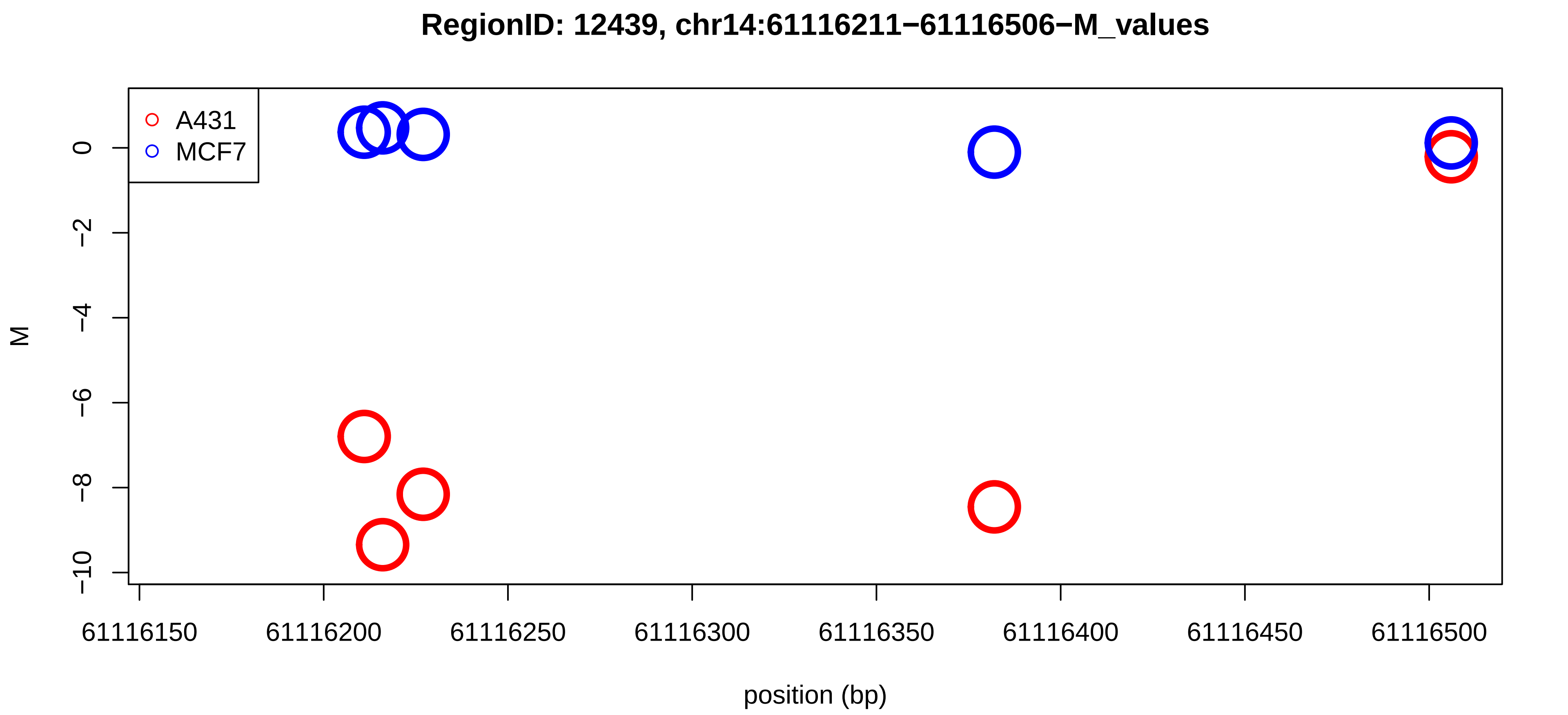

RegionID: 12439, chr14:61116211−61116506−M_values
A431
MCF7
M
0
−2
−4
−6
−8
−10
61116150
61116200
61116250
61116300
61116350
61116400
61116450
61116500
position (bp)

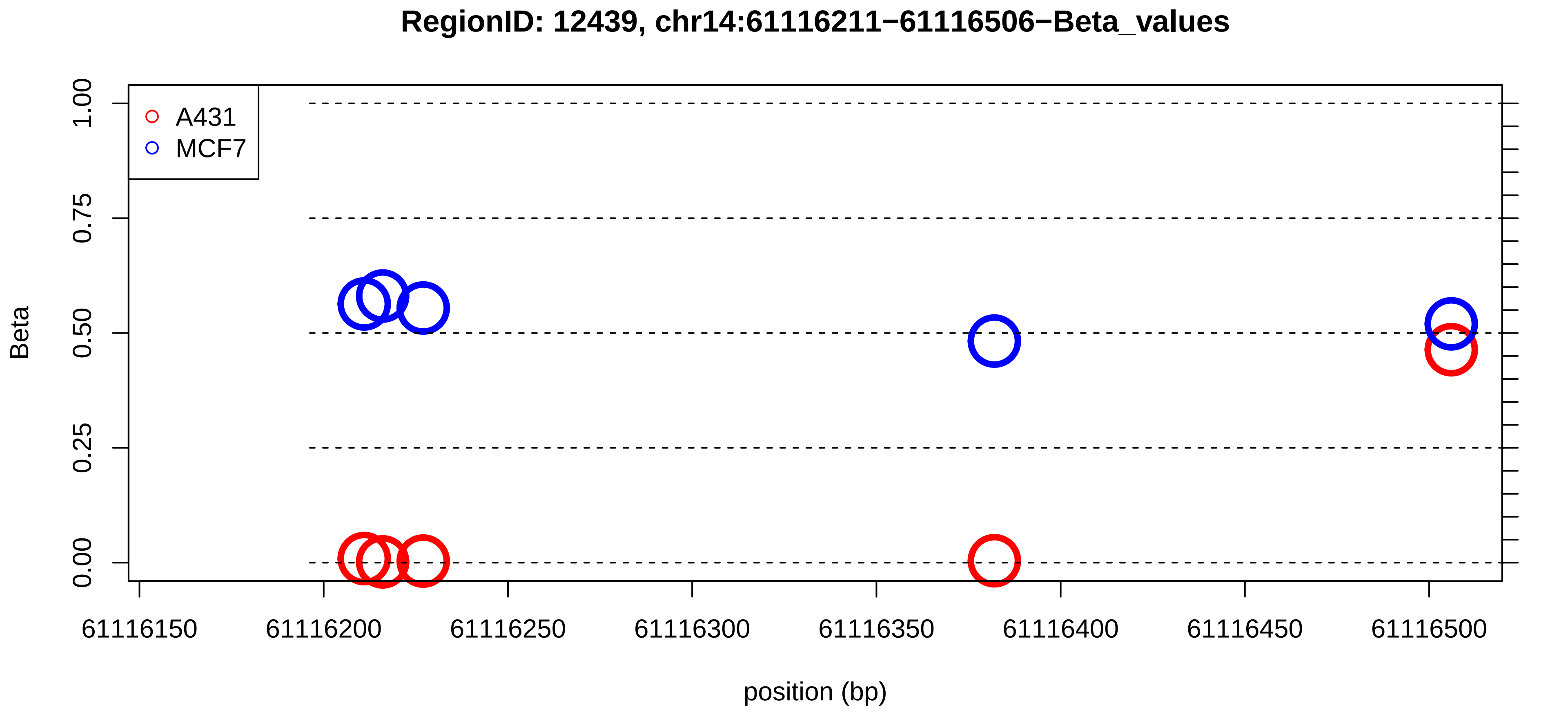

RegionID: 12439, chr14:61116211−61116506−Beta_values
A431
MCF7
Beta
0.00
0.25
0.50
0.75
1.00
61116150
61116200
61116250
61116300
61116350
61116400
61116450
61116500
position (bp)

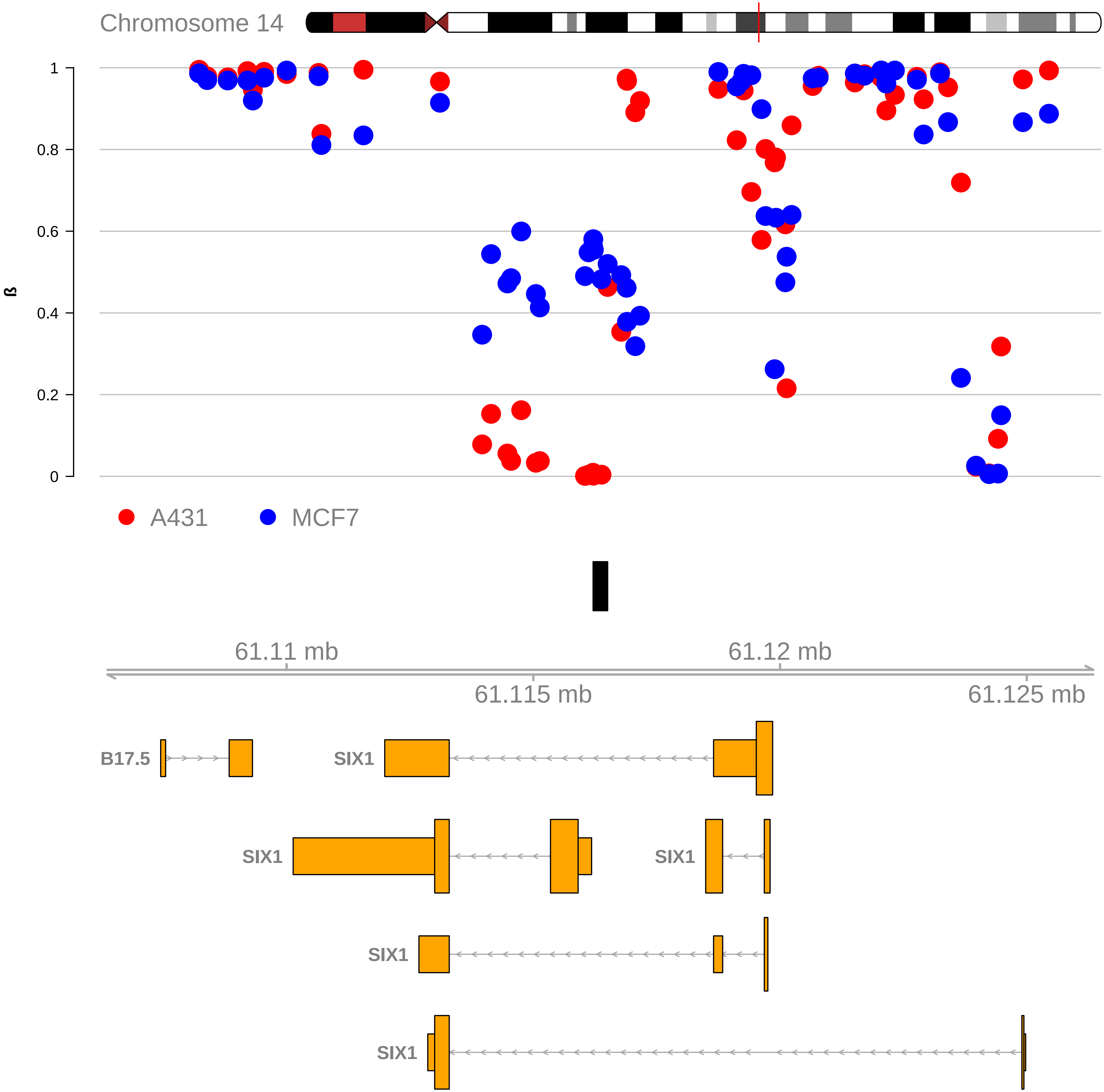

Chromosome 14
1
0.8
0.6
0.4
0.2
0
ß
A431
MCF7
61.11 mb
61.12 mb
61.115 mb
61.125 mb
B17.5
SIX1
SIX1
SIX1
SIX1
SIX1
